# Supplementary material for: Healthcare providers’ awareness of a mobile-based app for disease classification in northwest Ethiopia: A multilevel logistic regression analysis
Source: PLoS One. 2026 May 4;21(5):e0348362. doi: 10.1371/journal.pone.0348362 (PMC13138662; doi:10.1371/journal.pone.0348362)
Supplement: Attachment — Submitted filename: plos review.docx [file pone.0348362.s002.docx]

**First I would like to thanks to give me to put my expertise on this manuscript entitled”** **Healthcare providers’ awareness of a mobile-based app for disease classification in Northwest Ethiopia: A Multilevel Logistic Regression Analysis ”**

**General Question for the Couthers**

**As I have seen this manuscript is gives sound for the health sector. This is because currently Ethiopia uses ESV- ICD 11 and this is already launched and using practically so, what kinds of input dose this study add for those health care providers?**

**Specific Question**

1. **Do you think that currently is their M-based App functional in the health facility?**
2. **Form your regression you are using ICC how it could be? Do you think it is appropriate technique? Since your study participant were deferent levels**
3. **Form your result “Over half (55.73%) were aware with the ESV-ICD11's173 objective.” How this result could be? Because currently this system works for Medical doctors and HIT professionals, most of health care providers doesn’t have even information rather than awareness. Please clearly state this issue.**
4. **Have you seen the Category, Frequency and Percent from Table 2:** **Use ESV-ICD 11 booklet guide No = 37** a**nd Yes = 115 and Had challenge when using NHDD mobile app No = 44** a**nd Yes = 71 how this result is appeared make it clear?**
5. **From your result “Health care providers having social media accounts had substantially higher odds of awareness 207 (AOR = 13.96, 95% CI: 2.33–83.64, p < 0.01) than those without social media accounts.” could you interpreted the value of (AOR = 13.96, 95% CI: 2.33–83.64 the value of confidence interval and AOR is under question mark. I’m not clear such kinds of result.**
6. **From your Table 4: Multilevel Logistic regression analysis of Healthcare providers’ awareness of mobile-218 based app implementation for disease classification in Central Gondar Zone Healthcare: What is your outstanding reason to choice the reference? Make it scientific reason.**
7. **over half (55.73%) were aware with the ESV-ICD11's173 objective But From your conclusion one third of the health care provider were awarded based on your finding. How can you agree your report and your collusion please make it**

Tha**nk you for your interesting manuscript**
