# Supplementary material for: Healthcare providers’ awareness of a mobile-based app for disease classification in northwest Ethiopia: A multilevel logistic regression analysis
Source: PLoS One. 2026 May 4;21(5):e0348362. doi: 10.1371/journal.pone.0348362 (PMC13138662; doi:10.1371/journal.pone.0348362)
Supplement: Attachment — Submitted filename: Response to Reviewers.docx [file pone.0348362.s005.docx]

**Response to reviewers’ comments**

We would like to extend our sincere gratitude to the editor and reviewers for their time and commitment in providing informative and insightful comments on our manuscript titled “Healthcare providers’ awareness of a mobile-based app for disease classification in northwest Ethiopia: A Multilevel Logistic Regression Analysis." Your insights are greatly appreciated and have helped us improve the quality of our work.

Below, we outline our responses to the comments received. Thank you once again for your constructive suggestions.

**Reviewer 1 comments**

**Comment: First, I would like to thanks to give me to put my expertise on this manuscript entitled” Healthcare providers’ awareness of a mobile-based app for disease classification in Northwest Ethiopia: A Multilevel Logistic Regression Analysis.”**

***Author Response:*** *We want to start by saying thank you for reviewing our manuscript. Your questions and insights are helpful in making the work stronger. Our detailed responses to all of your concerns are listed below:*

**General Question for the Couthers**

**As I have seen this manuscript is gives sound for the health sector. This is because currently Ethiopia uses ESV- ICD 11 and this is already launched and using practically so, what kinds of input dose this study add for those health care providers?**

***Author response:*** *We appreciate your acknowledging the importance of this manuscript for the health sector. By filling up the existing gaps in healthcare professionals' knowledge and application of the ESV-ICD 11, the research findings improve Ethiopian healthcare delivery. Additionally, Ethiopian ministry of health jointly with Data use partnership (DUP) developed mobile-based ESV-ICD 11 app called the NHDD (National Health Data Dictionary) to enhance the accuracy of disease classification. Therefore, in our study, we tried to identify the current NHDD awareness level among healthcare providers and associated facility, individual level factors. Based on our findings, we recommend applying tailored interventions targeting on addressing influencing factors, improve awareness of health care providers, and ultimately implement NHDD app and guaranteeing improved disease classification.*

**Specific Question**

1. **Do you think that currently is their M-based App functional in the health facility?**

**Author response: Thanks for raising an important clarity question.** Yes, there is a mobile-based disease classification app known as NHDD (National Health Data Dictionary), which is a terminology service developed based on the open concept lab (OCL). Indeed, the NHDD app is made to work in healthcare facilities. Every healthcare provider can download the app in the app store since it is freely available. However, they download and use the app if they aware its existence. Therefore, our research try to identify gaps in healthcare providers' awareness and implementation of the app. The results show that improving accessibility and functionality will be crucial to the app's successful implementation into healthcare settings.

2. **Form your regression you are using ICC how it could be? Do you think it is appropriate technique? Since your study participant were deferent levels**

**Author response:** We appreciate the reviewer's thoughtful feedback. Since our multilevel logistic regression model takes into consideration the hierarchical structure of our data, it was suitable to apply the Intraclass Correlation Coefficient (ICC). Because healthcare providers are nested within healthcare facility levels, ICC had to be used in order to determine the variance related to these levels. The 19 facilities that made up the level-2 clustering unit in our study contained all healthcare providers. Contextual elements that may affect understanding of the mobile-based ESV-ICD-11 are shared among providers in the same facility, including management practices, culture, resources, and access to mentorship and training. The ICC measures clustering at the contextual level despite individual variances, which is comparable to frequent multilevel applications (e.g., patients within hospitals). According to methodological guidelines, the ICC indicates correlated observations within clusters, which supports multilevel modelling and displays the percentage of variance attributable to between-facility differences. While the ICC accounts for facility-level variation, the professional category is a covariate at the individual level.

3. **Form your result “Over half (55.73%) were aware with the ESV-ICD11's objective.” How this result could be? Because currently this system works for Medical doctors and HIT professionals, most of health care providers doesn’t have even information rather than awareness. Please clearly state this issue.**

***Author response: Thank you for raising an important issue. The ESV-ICD-11 is the nationally recognized disease classification system in Ethiopia, and columns for the ESV-ICD-11 diagnosis are included in most routine registers. Therefore, awareness of the ESV-ICD-11 list is essential for accurate documentation. For example, if a patient has both pneumonia and malaria, the doctor must enter only the primary diagnosis, either pneumonia or malaria, in the appropriate column. Not just physicians or HITs, but all healthcare professionals involved in patient care and documentation are required to use the ESV-ICD-11 system. Medical doctors are primarily located in hospitals, but nurses and health officers provide most clinical services in many health centers. To ensure the correct diagnosis is recorded in registers, these clinicians often refer to the ESV-ICD-11 list, either on paper or digitally.***

***Our study found that over half of respondents (55.73%) were aware of the purpose of the ESV-ICD-11. This suggests widespread use of the standard ESV-ICD-11 disease list, which replaced Ethiopia's previous HMIS diagnosis list. Additionally, our results show that 30.73% of providers knew about the NHDD mobile app, which provides the complete ESV-ICD-11 disease list electronically for reference. The ESV-ICD-11 disease list is available both in printed (paper) and in digital (NHDD app) formats, with the same information in both.***

4. **Have you seen the Category, Frequency and Percent from Table 2:** **Use ESV-ICD 11 booklet guide No = 37** a**nd Yes = 115 and Had challenge when using NHDD mobile app No = 44** a**nd Yes = 71? How does this result is appeared make it clear?**

***Author response:*** *I appreciate your thoughtful comments. Based on a total sample of 384 participants, we sought to evaluate the important components of ESV-ICD 11 in Table 2, such as the awareness of the ESV-ICD 11 manual (booklet) guide, the NHDD mobile app, and receiving mentorship or training.*

*To be clear, to determine the percentages shown in the table 2, we used the following method:*

***Awareness assessment:*** *First, we assessed participants' awareness of the ESV-ICD 11 booklet guide and the NHDD mobile app. For instance, of the 384 participants, 214 (55.73%) knew why they were using ESV-ICD 11, 152 (39.58%) knew about the ESV-ICD 11 booklet guide, and 118 (30.73%) knew about the NHDD mobile app.*

***Utilization assessment:*** *We asked people who knew if they had used the resources. In particular, 75.66% of participants reported using the booklet guide, with 115 out of 152 who were aware of it reporting doing so. Similarly, 58.47% of participants reported using the NHDD mobile app, with 69 out of 118 who were aware of it reporting doing so.*

***Challenge assessment****: Additionally, we particularly asked participants who had used the tools if they had any challenges. As a result, 50 participants (72.46%) experienced challenges with the NHDD mobile app, whereas 71 participants (61.74%) reported difficulties with the ESV-ICD 11 booklet-guide.
To improve the updated document's clarity, we have changed its wording to clearly state in table 2:*

- ***Use the ESV-ICD 11 booklet guide from those who are aware of it***
- ***Had a challenge when using the ESV-ICD 11 booklet guide from those who used it***
- ***Use of the NHDD mobile app by those who are aware of it***
- ***Had a challenge when using the NHDD mobile app from those who used it***

5. **From your result “Health care providers having social media accounts had substantially higher odds of awareness 207 (AOR = 13.96, 95% CI: 2.33–83.64, p < 0.01) than those without social media accounts.” could you interpreted the value of (AOR = 13.96, 95% CI: 2.33–83.64 the value of confidence interval and AOR is under question mark. I’m not clear such kinds of result.**

***Author response: Thank you for your understanding regarding the limitations associated with the wide confidence interval. We recognize that the 95% CI of (2.33 to 83.64) reflects variability due to our sample size. Out of 384 participants, only 59 had no social media accounts, and among them, only 2 were aware of the NHDD. Conversely, among the 325 participants with social media accounts, 116 reported awareness of the NHDD. This difference indicates that social media plays a significant role in information dissemination. In Ethiopia, the use of platforms like Telegram for sharing information has increased over time. The Ministry of Health, regional health bureaus, zonal health departments, district health offices, and health facilities have established various Telegram channels to share important information and gather feedback. For example, besides channels opened for general feedback and information sharing, there are specific channels for ESSV-ICD 11, such as: ESV ICD-11& MCCOD (opened at FMOH for use by all Ethiopian healthcare providers), ESV ICD 11 BDR R1 (Amhara region), ESV-ICD 11 (Oromia region), and others.***

***Given this context, the wide confidence interval reflects not only the limitations of our sample but also the strong association between social media account ownership and awareness of the NHDD.***

***We agree that the limited number of participants without social media accounts affects the robustness of our findings. Future research with a larger, more balanced sample could help further explore this relationship.***

***We appreciate your insights on this matter, as they help clarify the context and implications of our results.***

6. **From your Table 4: Multilevel Logistic regression analysis of Healthcare providers’ awareness of mobile-218 based app implementation for disease classification in Central Gondar Zone Healthcare: What is your outstanding reason to choice the reference? Make it scientific reason.**

***Author response:*** Thank you for your inquiry regarding the selection of reference categories in our multilevel logistic regression analysis of healthcare providers' awareness of mobile-based app implementation for disease classification.

*Our analysis's reference categories were chosen with statistical significance, representativeness, and clarity in consideration. For example, the majority in our sample is reflected when "Female" is used as a reference for gender, making it easy to understand how male providers differ. Comparably, "Diploma" was selected for educational status because it is common among healthcare professionals and allows for comparisons with holders of degrees and master's degrees. This strategy is consistent with other studies (e.g., Assaye et al., 2022) that use baseline categories for explaining their findings. By providing these references, we provide simple awareness comparisons between various groups, improving the usefulness of our findings in filling awareness gaps in the healthcare providers*.

7. **over half (55.73%) were aware with the ESV-ICD11's173 objective But From your conclusion one third of the health care provider were awarded based on your finding. How can you agree your report and your collusion please make it**

Tha**nk you for your interesting manuscript**

**Author response:** Thank you for your question. We are grateful for this chance to explain the confusing context of our results.

*The reported statistic of* 55.73% awareness *regarding the* ESV-ICD 11's objectives *indicates the understanding of why Ethiopia has introduced this new classification system, transitioning from the previous HMIS standard. It’s essential to note that while a significant portion of providers understand the objectives, some may still rely on the old HMIS system for recording, often inadvertently, due to insufficient training or information about the shift. Moreover, many healthcare providers may not have been adequately informed about whether their facilities had adopted the* ESV-ICD 11*, leading to continued use of outdated recording practices. With over* 2,913 disease classifications *identified in Ethiopia, memorizing all these diseases is challenging for providers, emphasizing the need for accessible reference tools. As a result, while* one third *of healthcare providers reported awareness of the mobile-based* NHDD app*, this statistic reflects their familiarity with practical tools designed to aid in disease classification. The discrepancy between these two figures illustrates the difference between understanding the objectives of ESV-ICD 11 and being informed about one of the specific resources available to implement these objectives effectively.*

**Reviewer 2 Comments**

I found this study very interesting, and I believe the outcomes have the potential to influence decision makers to facilitate and encourage wider use of mHealth applications among healthcare providers in Ethiopia. The findings highlight important gaps in awareness, and they can guide strategies to make such applications more accessible and integrated into clinical practice.

However, I would like to comment on two areas:

**Comment 1**

Study population: The study grouped together all healthcare providers, including nurses, doctors, technical assistants, and administrative staff. While I understand the intent to capture a broad perspective, it may not be entirely logical to include non-clinical staff, such as technical assistants and administrative personnel, since they are not the main target users of the NHDD mobile app. The analysis would be more focused and relevant if restricted to nurses and doctors, who are the primary groups that need—and should—use the application in daily practice.

**Author response:** Thank you for this insightful comment. We agree that the study population should reflect the primary users of the NHDD mobile application. In our setting, the included study participants: nurses, medical doctors, health officers, and health information technicians are all directly involved in disease classification and medical record documentation using the ESV–ICD-11 system at hospital and health center levels.

Health officers are clinical professionals who provide diagnostic and treatment services, primarily at health centers, and thus function similarly to medical doctors at the hospital level. They are not administrative staff. Health information technicians, while not providing direct patient care, are responsible for recording diagnoses from patient charts and recording them, using ESV–ICD-11 in outpatient and inpatient registers.

In many facilities, nurses also perform this classification and coding role based on clinicians’ documentation from patient charts. Importantly, we excluded staff who are not primarily responsible for disease classification (e.g., laboratory technicians, pharmacists, environmental health professionals). Although some of these professionals may transcribe already-classified diagnoses into their respective registers, they do not perform the initial ESV–ICD-11 classification and are therefore not primary users of the application. Because responsibility for ESV–ICD-11 recording varies across facilities, restricting the study population to only doctors and nurses would not fully capture real-world use of the NHDD mobile application. We therefore believe that including all healthcare providers who directly perform ESV–ICD-11 classification and coding provides a more accurate and contextually appropriate assessment of the NHDD app’s usability and adoption.

**Comment 2**
Writing style: The overall clarity of the manuscript would benefit from further editing, particularly in the Results section. The presentation of findings sometimes lacks flow and precision, which may make it difficult for readers to follow the main outcomes. A clearer, more concise reporting of the statistical results would strengthen the impact of the paper.

**Author response:** We are grateful for this constructive feedback. The manuscript has undergone thorough editing to enhance its accuracy, flow, and clarity, especially in the Results section. To make the key findings easier to follow and understand, we refined the presentation of findings, minimized duplication, and clarified the reporting of statistical results. We think these changes have improved the manuscript's overall impact and readability.
