# Supplementary material for: Healthcare providers’ awareness of a mobile-based app for disease classification in northwest Ethiopia: A multilevel logistic regression analysis
Source: PLoS One. 2026 May 4;21(5):e0348362. doi: 10.1371/journal.pone.0348362 (PMC13138662; doi:10.1371/journal.pone.0348362)
Supplement: Attachment — Submitted filename: comment.docx [file pone.0348362.s004.docx]

Thank you for addressing the comment. Better to address all the comments to increases the quality of the paper. Gramer, coherence and discussion part

Thank you
